# A dynamic panel threshold model analysis on heterogeneous environmental regulation, R&D investment, and enterprise green total factor productivity

**DOI:** 10.1038/s41598-024-55970-1

**Published:** 2024-03-03

**Authors:** Lu Liu, Rong Ren, Kaiyuan Cui, Lei Song

**Affiliations:** 1 School of International Trade and Economics, Shandong University of Finance and Economics, Jinan, 250014 China; 2 School of Management, Shandong University, Jinan, 250100 China; 3 School of Economics and Management, Shandong Youth University of Political Science, Jinan, 250103 China; 4 School of Economics, Ocean University of China, Qingdao, 266100 China

**Keywords:** Environmental regulation, Enterprise green total factor productivity, R&D investment, Digital level, Panel threshold model, Environmental economics, Sustainability

## Abstract

Environmental regulations are important means to influence manufacturing enterprise green development. However, there are two completely different conclusions both in theoretical and in empirical research, namely the “Follow Cost” theory and the “Porter Hypothesis”. The nonlinear mechanism needs to be considered. Therefore, this study aims to explain the threshold impact of heterogeneous environmental regulations on enterprise green total factor productivity. Environmental regulations are divided into different sub-categories, then based on the panel data of 1220 Chinese manufacturing listed companies from 2011 to 2020, this paper uses threshold regression model to examine the impact of heterogeneous environmental regulations on Chinese manufacturing enterprise Green Total Factor Productivity. The empirical results show that: (1) Command-controlled, market-incentive and voluntary-agreement environmental regulation all have a significant nonlinear impact on enterprise Green Total Factor Productivity. (2) Enterprise R&D investment plays a threshold role in the impact. (3) There are industry and equity type differences in the impact process. This study focuses on the micro level of enterprises and tests the threshold mechanism, which make some theoretical complement to previous researches. The research results are not only beneficial for the government to propose appropriate environmental regulatory policies, but also for enterprises to achieve green growth through heterogeneous R&D investment.

## Introduction

Since the implementation of the reform and opening-up policy in 1978, China’s economy has developed rapidly. However, the development of Chinese economy has occurred at the expense of the environment^1,2^. That is, the traditional extensive economic development mode characterized by “high growth and high energy consumption” has increased the burden on China’s ecological environment, further led to increasingly prominent environmental problems, and become an obstacle to the economy’s sustainable development^3^. According to the BP World Energy Statistical Yearbook (2022), in 2021, CO_2_ emissions generated by energy in China are 10.523 billion tons, accounts for 31.06% of the whole world, far more than other regions. At the same time, the global economy has shown the trend of low-carbon emission and green sustainable development. The implementation of the Paris Agreement has further established a clear agenda for global carbon reduction. Therefore, carbon emissions reduction is an important and arduous task both for environmental remediation in China and for promoting green development throughout the world^4^. GTFP, as a combination strategy under the dual goals of economic development and environmental protection, helps to promote economy’s sustainable development.

Manufacturing enterprises are both micro entities and carbon emitting entities of the national economy. According to the Carbon Emission Ranking of Chinese Listed Companies, the total carbon emissions of the 100 listed companies in 2022 were 5.046 billion tons, accounting for approximately 45.87% of China’s total carbon emissions. Obviously, manufacturing enterprises play an important role in achieving green and sustainable development. Green total factor productivity (GTFP) takes into account energy loss and pollution emissions in the production process, and is a comprehensive indicator for measuring economic performance and ecological environment performance. GTFP of manufacturing enterprises is the core and starting point for achieving broader economic and environmental goals. Therefore, it is of great significance to enhance GTFP of Chinese manufacturing enterprises^5^.

Enterprises are profit oriented economic organizations, the market economy, as a powerful engine of human development, has important imperfections^6^, thus relying solely on the invisible hand of the market is difficult to promote its spontaneous green production. Government policies play an important role in promoting sustainable technological progress and environmental sustainability^7,8^. Environmental regulations have important impacts on manufacturing enterprises GTFP^9–11^ Thus, the Chinese government has utilized heterogeneous environmental regulations to promote enterprises green development. On one hand, a series of command-controlled environmental regulations have been made, including the Environmental Protection Law of China, the Cleaner Production Promotion Law in China, Atmospheric Pollution Prevention and Treatment Law in China etc. On the other hand, some market-incentive environmental regulations such as carbon emission quotas are used to encourage manufacturing enterprises green development.

Governments around the world actively formulate environmental regulations, hoping to promote green development. And related researches on the impact of environmental regulations on GTFP has attracted great attention. However, there are two completely different views both in theoretical and empirical, “the driving role of environmental regulations” and “the hindering role of environmental regulations”^12–14^. According to the “Follow Cost” theory, the stricter environmental regulations are, the higher cost of enterprises pollution controls are, which will furthermore restrain the production efficiency and profitability of enterprises, and hinder the improvement of enterprises GTFP^15^. However, according to the theory of “Porter Hypothesis”, moderate environmental regulations can encourage enterprises to engage in more innovative activities, which will increase their productivity, offset the investment cost caused by environmental regulations and enhance their profitability in the market^16,17^.

Empirical research also has completely different conclusions. Some empirical results have found that environmental regulations can improve both industry production efficiency and environmental performance by influencing technological progress, thereby achieving green growth^18^. Some other empirical results have showed that environmental regulations have caused cost increases, and their contribution to technological innovation is relatively small or even inhibitory, which is not conducive to the improvement of production efficiency and environmental performance, and thus hinders green growth^19^. There are also some empirical researches have confirmed that there is a non-linear relationship or even no correlation between environmental regulations and green development^16^.

Theoretical and empirical research have not yet reached a consensus, and there are two possible reasons. Firstly, the existing researches on the impact of environmental regulations on green growth mainly focus at the regional or industry level, related researches at the level of enterprise is insufficient^15^. In fact, both the “Following Cost” theory and the “Porter Hypothesis” have their rationality, and the reason of contradiction lies in the coexistence of cost increase and innovation compensation caused by environmental regulations, but there is insufficient discussion on the boundary conditions. As heterogeneous individuals, enterprises may have quite different reactions to the same environmental regulatory policies. And only the exploration that focuses on the micro level of enterprises may open this “black box”. Secondly, environmental regulations are a comprehensive concept that can be further divided into different subcategories, which may lead to inconsistent research conclusions if confusing them together. Therefore, it is necessary to explore the mechanism of environmental regulations affecting GTFP of heterogeneous enterprises at micro level. Thirdly, both theoretical and empirical researches have shown that the impact of environmental regulations on green development may be non-linear. Environmental regulations only generate external conditions that can affect enterprises’ behavior. Whether and to what extent an enterprise innovates is determined by enterprise heterogeneity factors, and R&D investment is an important determining factor. Therefore, this article uses enterprise R&D investment as a threshold variable to explore the threshold impact of heterogeneous environmental regulations on enterprise GTFP.

**Figure 1:**
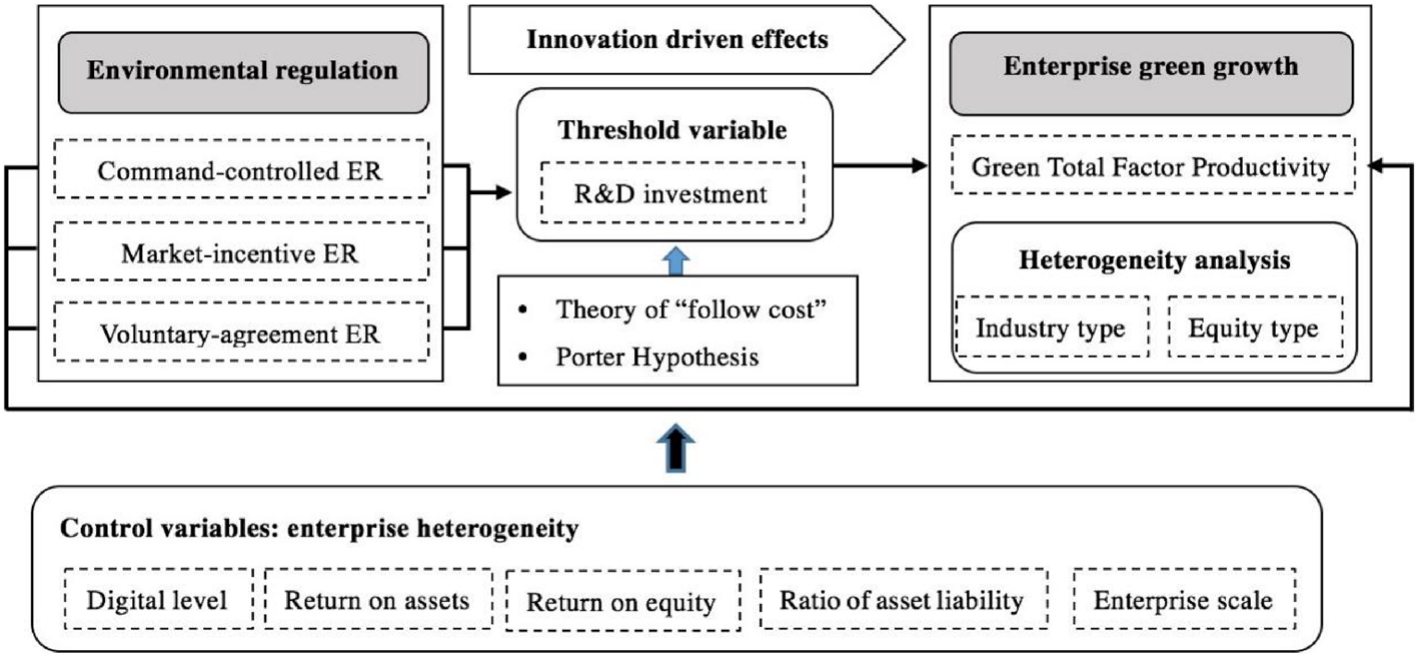
Analytical framework of this study.

The contribution of this paper mainly lies in the following: (1) Focusing on the micro level of enterprises, this paper divides environmental regulations into three heterogeneous subcategories including command-controlled type, market-incentive type and voluntary-agreement type, and furthermore clarify the mechanism of heterogeneous environmental regulations affecting enterprise GTFP; (2) Focusing on the micro level of enterprises, enterprise R&D investment is introduced as the threshold variable to clarify the panel threshold mechanism and determine the threshold values; (3) Heterogeneity analysis is conducted based on enterprise industry type and enterprise equity type. The research conclusions can provide theoretical reference and decision-making reference both for enterprises from micro level and for policy makers from macro level. The research framework of this paper is illustrated in Fig. 1.

## Literature review and hypothesis

### Heterogeneity environmental regulations and enterprise GTFP

The research on the impact of environmental regulations on GTFP has become a fore issue, and different impact direction of the empirical results have also spawned further thinking of scholars. Firstly, researchers propose that environmental regulations should be divided into different subcategories, and the impact of heterogeneous environmental regulations on GTFP are different^20,21^. Researchers conducted deeper discussions on heterogeneous environmental regulations, and most agree that environmental regulation can be divided into three subtypes, which are command-controlled environmental regulation (CER), market-incentive environmental regulation (MER) and public-participation environmental regulation (VER)^22^. Secondly, the existing researches mainly focuses on the regional and industry levels, and there is still insufficient research on the enterprise level^15^. Thirdly, limited researches on enterprise green growth mainly focuses on enterprise green patents. On the one hand, this calculation doesn’t consider input–output ratio can easily lead to biased research conclusions. On the other hand, it cannot verify the impact mechanism on many enterprises without green patents. The traditional methods used to measure productivity growth ignore the pollutants that are produced by the production process, while GTFP takes into account pollutant emissions during the production process, and measures enterprise green growth from the perspective of input-output^7^. Thus, GTFP can not only include all enterprises but also better reflect enterprise green growth efficiency.

Undoubtedly, environmental regulation is one of the most important forces in achieving green growth^23^. Command-controlled environmental regulation (CER) refers to the government guiding and standardizing the production process of enterprises through a series of administrative means, such as environmental regulations, rules, policies, emission standards and so on. On the one hand, the strict CER given by the government will inevitably increase enterprise environmental governance costs and then lead to excessive environmental governance expenditures in the short term, which is unfavorable for enterprise green growth^24^. The research utilizing a comprehensive CS-ARDL model and using data of OECD countries from 1990 to 2020 found that environmental policies are effective in reducing carbon dioxide emissions^25^. The empirical study based on the panel data of China’s 31 energy-mineral cities in 2007–2018 showed that CER has an inhibitory impact on energy eco-efficiency, and the inhibitory effect is more obvious in central and northeastern regions^26^. Research adopted the Spatial Durbin Model found that CER will hinder regional green technology innovation^27^. On the other hand, CER will significantly increase enterprise environmental legal costs of production and operation process, thereby playing a deterrent role and help to promote enterprise technological innovation, and achieving green growth^15^. Based on panel data from 30 provinces in China from 2003 to 2017, the results of Systematic Generalized Method of Moments indicated that CER has a significant promoting effect on green innovation^28^. The empirical study on the data of Chinese A-share companies listed from 2010 to 2019 found that CER can stimulate enterprise green technology innovate^29^. Besides, CER may has a nonlinear impact on green development. Some research suggested that CER has a significant threshold impact on green technological innovation^30^, and when its intensity exceeds a certain threshold, green technology innovation is improved. Similar researches found that with the improvement of economic level, environmental regulation shows a “U” relationship of first restraining and then promoting technological innovation^31^.

From the above analysis, it can be seen that both theoretical and empirical studies have not reached a consensus on CER’s impact on GTFP, and threshold effect may exist. Thus, Hypothesis 1 is proposed.

#### Hypothesis 1

CER has a threshold effect on enterprise GTFP.

Market-incentive environmental regulation (MER) mainly refers to market-oriented institutional regulatory measures especially economic means such as pollution control investment, environmental protection fund investment and other tax standards, aim to guide enterprises achieving green production^32^.

Theoretical research has not reached a consensus on MER’s impact on GTFP. On the one hand, there is a divergence between the “Follow Cost” theory and the “Porter Hypothesis”. On the other hand, even the “Porter Hypothesis” suggested that only those carefully designed environmental regulations can promote green development by enhancing innovation. That is, the mechanism between carefully designed regulation and its potential innovation offsetting effects is not yet clear^33^.

The empirical research results are also quite different. Gray and Shadbegian found that higher pollution abatement costs significantly decrease the productivity^34^. According to the panel data of A-share new energy companies listed in Shanghai and Shenzhen from 2012 to 2020, the research drew the conclusion that MER could promote the green innovation of new energy firms^35^. Based on the data of 30 provinces in China during the period of 2000 to 2012, the empirical results showed that environmental expenditure as one type of MER has a U-shaped impact on green productivity growth of China’s industry^15^. Some scholars pointed that when the intensity of MER is not high, the environmental cost of enterprises is relatively low, so enterprises lack the power of technological innovation and thus prevent the improvement of GTFP, when the intensity of MER is high enough, enterprises have a strong driving force for technological innovation, and then promote the improvement of GTFP^36,37^. Based on the above analysis, the impact of MER on GTFP is relatively complex and threshold effect may exist. Thus, Hypothesis 2 is proposed.

#### Hypothesis 2

MER has a threshold effect on enterprise GTFP.

Voluntary-agreement environmental regulation (VER) also known as public-participation based environmental regulation, refers to the regulation of corporate behavior through external supervision by individuals or non-governmental organizations, such as the number of environmental petitions, batches of environmental petitions, and the number of environmental petitions. The public could actively perform duties of environmental protection through environmental letters or visits and media supervision, reflect enterprise environmental pollution problems to the government, so as to supervise enterprise pollution behaviors^38^. Therefore, it conveys an implicit message to the outside world, that is, the enterprise has social responsibility and pays attention to environmental protection^33,39^. A good corporate image in turn helps enterprise attract more external investment and provides support for its green technological innovation. According to the research of Jia^40^, the greater VER is, the more enterprises will be encouraged to take more measures to pursue environmental performance, and therefore has a positive impact on GTFP. Taking A-shared listed enterprises in China from 2010 to 2019 as samples, the empirical results showed that VER inhibited enterprise green technological innovation^29^. Based on panel data of 86 Chinese steel enterprises from 2005 to 2014, the paper analyzed that VER has a direct and positive effects on the enterprise technological innovation^41^.

Based on the above analysis, the impact of VER on GTFP may exist a threshold effect. Thus, Hypothesis 3 is proposed.

#### Hypothesis 3

VER has a threshold effect on enterprise GTFP.

### R&D investment and enterprise GTFP

The important mechanism by which environmental regulations affect enterprise GTFP is to promote the internalization of enterprise environmental governance costs, so as to stimulate enterprise innovation activities, and then affect enterprise GTFP^6^. However, it is still unclear for the mechanism between the cost increase caused by environmental regulations and possible innovation compensation^33^. The heterogeneity of enterprise, especially enterprises differentiated innovation level became an important determining factor that affects the driving effect. R&D investment is an important indicator to measure the level of innovation^6^. The environmental regulations’ driving effect is mainly achieved by influencing enterprise R&D investment. Therefore, it is necessary to further clarify the impact of enterprise R&D investment on GTFP from the enterprise level.

According to the Theory of Endogenous Growth, innovation can form a new production function, and technological progress driven by knowledge spillovers and knowledge transfer is the decisive factor for sustainable growth^6^. R&D investment is the source of enterprise innovation and economic growth. R&D investment is conducive to optimizing production factors and reducing information asymmetry, this not only expand the source of explicit knowledge, but also increase organization flexibility, which can further promote the spillover and absorption of tacit knowledge, and then improve enterprise performance. R&D promotes environmental protection by reducing CO_2_ emissions^25^. Based on the panel data of high-tech enterprises in China from 2012 to 2017, the research proposed that innovation investment plays a mediating role in the impact of heterogeneous environmental regulations on enterprise innovation^42^.

The “Follow Cost” theory suggests that environmental regulations will lead to an increase in enterprises pollution control costs. Especially in the short term, constraints by capital amounts, enterprises have to increase the pollution discharge investment which will inevitably lead to a decrease in R&D investment and resulting “R&D Crowding Out Effect”^43^. However, the “Porter Hypothesis” suggests that in the long term, reasonable and strict environmental regulations can promote technological practices. The costs of environmental regulations may be partially or entirely offset by the compensating effects of innovation activities, and thus environmental regulations can promote GTFP through the “Innovation Compensation Effect”^44^.

The magnitude of the “R&D Crowding Out Effect” and “Innovation Compensation Effect” affects the performance of environmental regulatory policy. Enterprise R&D investment is an important factor affecting R&D costs and long-term technological innovation capabilities, and may have a significant impact on heterogeneous environmental regulations affecting enterprise green development^29^.

The continuous developing of relevant research has laid the foundation and provided a new perspective for further exploring the impact of heterogeneous environmental regulations’ innovation driving effect. Therefore, this paper introduces enterprises R&D investment in the analysis, and believes that enterprises R&D investment plays an important role in the impact of heterogeneous environmental regulations on enterprises GTFP^45,46^.

Based on the above analysis, Hypothesis 4, Hypothesis 5 and Hypothesis 6 are proposed.

#### Hypothesis 4

Enterprise R&D investment plays a threshold role in the impact of CER on enterprises GTFP.

#### Hypothesis 5

Enterprise R&D investment plays a threshold role in the impact of MER on enterprises GTFP.

#### Hypothesis 6

Enterprise R&D investment plays a threshold role in the impact of VER on enterprises GTFP.

## Research methodology

### Sample selection

The samples of this paper are panel data of 1220 A-share manufacturing listed companies in China. The relevant data is obtained from the China Stock Market and Accounting Research Database (CSMAR). Firstly, according to the industry classification codes in the Guidelines for Industry Classification of Listed Companies (2012 Revision) issued by the China Securities Regulatory Commission, 31 categories of A-share manufacturing listed companies are selected. Secondly, excluded enterprises that have not been listed or have been delisted during the study period, excluded enterprises that have been ST、*ST or samples with lots of missing data. Finally, 12,200 pieces of panel data of the sample enterprises from 2011 to 2020 are obtained.

### Variables measurement and data sources

#### Dependent variables

The dependent variable of this paper is sample enterprises GTFP. Scholars have used several methods to measure green productivity issues, such as descriptive analysis, non-parametric analysis including data envelope analysis, input–output analysis, dynamic computable general equilibrium, parametric analysis based on a combined method of parametric analysis^7^. This article constructs the optimal frontier of enterprises green growth based on the Super-SBM Model includes undesirable outputs, and calculates enterprises GTFP according to the Super-SBM Mode^47–49^. Specifically, the measurement of GTFP involves input factors, desirable output, and undesirable output.

Firstly, referring to related methods for measuring GTFP, this paper selects input indicators includes capital input, labor input, intermediate input, and energy input^4,50^. Referring to the general method, enterprise capital input is calculated by “the perpetual inventory method” based on the “net value of enterprise fixed assets”, enterprise labor input is calculated by “the cash paid by the enterprise to employees”, enterprise intermediate investment is calculated by “the sum of operational expense, sales expenses, financial expenses, and management expenses minus the cash paid to and for employees, and minus depreciation and amortization”, enterprise energy investment is calculated by “the proportion of enterprise gross output value to the gross industry output value, and multiply the energy consumption of the industry that enterprise belongs to”. Secondly, enterprise desirable output is calculated by “enterprise main business income”. Thirdly, enterprise undesirable output is calculated by “three kinds of enterprise’s industrial waste”, including the industrial wastewater, SO2, and industrial smoke emissions of enterprise. The relevant data comes from CSMAR, China Statistical Yearbook, China Environmental Statistical Yearbook, and China Urban Statistical Yearbook.

#### Independent variable

The independent variable of this article are heterogeneous environmental regulations, which includes three subcategories: CER, MER and VER. Referring to current calculation method, CER is calculated by “the number of environmental case proposals submitted by provincial and municipal people’s congresses”^26^. Investment of environmental pollution control as one of the MER method, can demonstrate the incentive costs invested by regional governments and continuous data can be obtained. Therefore, MER in this article is calculated by “the ratio of the completed investment in industrial pollution control in each region to the regional GDP” ^50–53^. Considering previous research and data availability, VER is calculated by “the number of environmental petitions in each province and city”^29^. Relevant data are collected from China Environmental Statistical Yearbook.

#### Threshold variables

The threshold variable of this article is enterprise R&D investment (RD). Firstly, there is a close correlation among environmental regulations, R&D investment and GTFP. According to Porter Hypothesis, environmental regulations have innovation driven effects. In fact, environmental regulations only generate external conditions that can affect enterprises’ behavior. Whether and to what extent an enterprise innovates is determined by enterprise heterogeneity factors, and R&D investment is an important determining factor. Secondly, enterprise R&D investment can be clearly measured. Thirdly, data of R&D investment is reliable. R&D investment can be found in the annual reports of listed companies. The disclosure of this indicator is very comprehensive, with few missing items, and the data is available and reliable. Therefore, enterprise R&D is used this article investment as a threshold variable to explore the threshold impact of heterogeneous environmental regulations on enterprise GTFP. Enterprise R&D investment is measured by the proportion of enterprise R&D amount in sales revenue^35^. Relevant data is from CSMAR.

#### Control variables

Referring to relevant literatures on environmental regulations and enterprise green growth, the following indicators are selected as control variables^54–56^: enterprise digital level (DL), return on assets (ROA), return on equity (ROE), ratio of asset liability (ROL), and enterprise scale (Scale). Enterprise digital level is measured by “the number of digitization-related-words frequencies in enterprise annual reports” based on Python word frequency statistical analysis methods. Enterprise return on assets is measured by “the proportion of enterprise’s net profit in the total assets”. Enterprise return on equity is measured by “the proportion of enterprise’s net profit to net assets”. Enterprise ratio of asset liability is measured by “the proportion of its total liabilities to total assets”. Enterprise scale is measured by “enterprise total assets”.

#### Grouping variables

In order to test whether there is heterogeneity in the threshold regression results, referring to classic theories and considering enterprise most important heterogeneity, enterprise industry (Indus) and enterprise equity (Equ) type are selected as two grouping variables. Enterprise industry can be divided into three categories based on their pollution emission levels which are high pollution industry, medium pollution industry and low pollution industry. Enterprise equity can be divided into two categories based on the equity status which are state-owned and non-state-owned.

All the variables except for grouping and ratio variables are logarithmically processed after adding one. And the summary of variables is shown in Table 1.

**Table 1:** The summary of variables.

Variable	Abbreviation	Description
Dependent variables	GTFP	Input factors, desirable output, undesirable output; Super-SBM Model
Independent variables	CER	The number of environmental case proposals submitted by provincial and municipal people’s congresses
	MER	The ratio of the completed investment in industrial pollution control in each region to the regional GDP
	VER	Number of environmental petitions in each province and city
Threshold variables	RD	The proportion of enterprise R&D amount in sales revenue
Control variables	DL	The number of digitization-related-words frequencies in enterprise annual reports
	ROA	The proportion of enterprise’s net profit in the total assets
	ROE	The proportion of enterprise’s net profit to net assets
	ROL	The proportion of its total liabilities to total assets
	Scale	Enterprise total assets
Grouping variables	Indus	Industry pollution level
	Equ	Enterprise equity type

### Model construction

Firstly, according to literature review, it was found that environmental regulations have both promoting and inhibiting effects on green development. The current research debate focuses on the conditions for a reasonable explanation of these two effects. Secondly, threshold regression models are non-linear models that are suitable for explaining the non-linear relationships between variables, especially the threshold effect that both positive and negative effects exist simultaneously. Thirdly, this article attempts to explore the threshold impact of heterogeneous environmental regulations on enterprise GTFP. The threshold regression model is very suitable to explain the mechanism that this article proposed.

Based on all the above theoretical analysis, in order to test the research hypotheses H1-H6 and find the optimal range of R&D investment that can maximize enterprise GTFP, the panel threshold model based on Hansen model^57^ is shown as Eq. (1). In the equation, GTFP_*i,t*_ represents enterprises green total factor productivity, I (*) is the indicator function, enterprise R&D investment is taken as the threshold variable and* γ*_*1*_、*γ*_*2*_ is the threshold of threshold of the effect. *α*_*0*_ is constant terms, *α*_*1*_*、α*_*2*_*、α*_*3*_*、ρ*_*i*_ are the regression parameters, *θ*_*i*_ is the unobservable individual effect, ε_*i,t*_ is the random error term. Other variables are the same as the above.

1 $$\begin{aligned} GTFP_{i,t} & = \propto_{0} + \propto_{1} ER_{i,t} I\left( {RD_{i,t} \le \gamma_{1} } \right) + \propto_{2} x_{i,t} I\left( {\gamma_{1} < RD_{i,t} \le \gamma_{2} } \right) \\ & \quad + \propto_{3} x_{i,t} I\left( {RD_{i,t} > \gamma_{2} } \right) + \rho_{{\text{i}}} Control_{i,t} + \theta_{i} + \varepsilon_{i,t} \\ \end{aligned}$$

## Results and analysis

### Description and correlation analysis

The descriptive statistics of variables and the correlation analysis results are shown in Table 2. It can be seen from the table that the average values of GTFP is 0.698, reacting that many sample enterprises GTFP is not very high, the standard deviation of GTFP is 0.184, indicating that small differences in GTFP among sample enterprises. Among the three subcategory tools for environmental regulations, the standard deviation of MER is the minimum and the standard deviation of VER is maximum. In addition, the variance of most variables is less than the mean value, indicating that the dispersion coefficient is relatively small and the stability of the sample is good. The Pearson correlation analysis results are also shown in Table 2. It can be seen that there is interdependence between variables, but it cannot distinguish the causal relationship of variables. Therefore, it is both possible and necessary to further establish quantitative relationships between variables through regression analysis.

**Table 2:** The descriptive statistics and correlation coefficients of variables.

Variables	1	2	3	4	5	6	7	8	9	10
GTFP	1.000									
CER	− 0.005	1.000								
MER	− 0.006	− 0.242***	1.000							
VER	− 0.033***	0.577***	− 0.356***	1.000						
RD	0.028***	0.024***	− 0.083***	0.075***	1.000					
DL	− 0.032***	0.065***	− 0.167***	0.161***	0.265***	1.000				
ROA	− 0.002	0.001	− 0.007	0.005	− 0.017*	− 0.003	1.000			
ROE	− 0.007	− 0.018*	0.003	− 0.006	− 0.002	− 0.013	0.827***	1.000		
ROL	− 0.020**	− 0.023**	− 0.040***	− 0.021**	− 0.155***	− 0.040***	− 0.056***	0.019**	1.000	
Scale	− 0.092***	− 0.044***	0.030***	− 0.013	− 0.110***	0.187***	− 0.022**	-0.033***	0.238***	1.000
Mean	0.698	6.491	0.733	8.511	4.010	1.608	3.683	13.784	42.364	22.193
Std	0.184	0.787	0.273	1.112	3.993	1.064	24.739	729.767	31.097	1.230

***, ** and * indicate the statistical significance of 1%, 5% and 10% respectively.

### Threshold effect test

**Figure 2:**
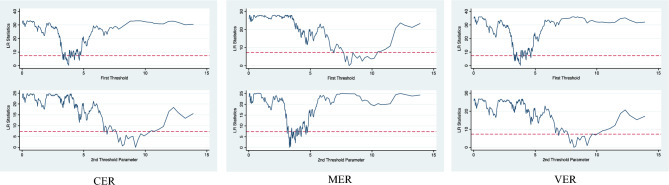
Double threshold effect diagram of the impact of heterogeneous environmental regulations on GTFP.

Firstly, examine whether the threshold effect exists and determine the number of thresholds. The results are shown in Table 3. According to the results, enterprise R&D investment is the threshold variable, CER, MER and VER all have significant dual threshold effects on enterprise GTFP. Double threshold effect diagram is shown in Fig. 2.

**Table 3:** Threshold Test Effect Results.

ER type	Threshold type	Threshold	95% Conf. Interval	F	*p*
CER	Single	3.7900***	[3.635, 3.800]	37.03	0.0000
	Double	9.2100***	[8.290, 9.420]	24.76	0.0000
	Triple	–	–	9.96	0.5133
MER	Single	8.140 0***	[7.750, 8.290]	32.60	0.0000
	Double	3.3000***	[3.250, 3.320]	25.12	0.0000
	Triple	–	–	9.23	0.7033
VER	Single	3.7900***	[3.635, 3.800]	41.67	0.0000
	Double	8.1400***	[7.775, 8.290]	26.89	0.0000
	Triple	–	–	9.68	0.7600

***, ** and * indicate the statistical significance of 1%, 5% and 10% respectively.

Further threshold regression analysis was conducted on three types of heterogeneous environmental regulations, and the overall results are shown in Tables 4, 5, and 6.

**Table 4:** Threshold regression results of CER on enterprise GTFP.

	Overall GTFP	Industry 1 GTFP	Industry 2 GTFP	Industry 3 GTFP	State GTFP	Non-state GTFP
RD < r_1_	0.0072 (1.31)	0.0237** (2.04)	− 0.0069 (− 0.77)	0.0040 (0.51)	0.0071 (0.77)	− 0.0074 (1.05)
r_1_ ≤ RD < r_2_	0.0119** (2.18)	−	−	0.0089 (1.16)	−	0.0122* (1.73)
RD ≥ r_2_	0.0203*** (3.63)	0.0302** (2.55)	− 0.0010 (− 0.11)	0.0226*** (2.64)	0.0145 (1.59)	0.0243*** (3.18)
DL	0.0121*** (4.57)	0.0209*** (3.75)	0.0078 (1.52)	0.0107*** (3.02)	0.0145*** (3.03)	0.0116*** (3.67)
ROA	− 0.0000 (− 0.03)	0.0007 (1.36)	− 0.0003 (− 0.69)	− 0.0024*** (− 2.87)	0.0005 (1.30)	− 0.0013* (− 1.63)
ROE	− 0.0000 (− 0.17)	− 0.0000 (− 0.29)	0.0000 (0.63)	− 0.0000*** (− 4.76)	− 0.0000* (− 1.72)	0.0000* (1.67)
ROL	− 0.0001 (− 0.63)	0.0005 (1.09)	0.0000 (0.50)	− 0.0004*** (− 3.77)	0.0009** (2.17)	− 0.0002 (− 1.39)
Scale	− 0.0629*** (− 11.66)	− 0.0620*** (− 5.30)	− 0.0648*** (− 6.53)	− 0.0604*** (− 8.65)	− 0.0687*** (− 6.36)	− 0.0594*** (− 9.98)
cons	2.0173*** (17.78)	1.8725*** (7.39)	2.1571*** (10.37)	1.9889*** (13.81)	2.1240*** (9.50)	1.9340*** (15.12)
R^2^	0.0292	0.0317	0.0339	0.0447	0.0353	0.0326
F	28.63***	6.98***	10.95***	28.80***	13.41***	21.78***

***, ** and * indicate the statistical significance of 1%, 5% and 10% respectively.

**Table 5:** Threshold regression results of MER on enterprise GTFP.

	Overall GTFP	Industry 1 GTFP	Industry 2 GTFP	Industry 3 GTFP	State GTFP	Non− state GTFP
RD < r_1_	− 0.0460*** (− 3.98)	− 0.3511* (− 1.60)	0.0076 (0.40)	− 0.0879*** (− 4.48)	− 0.0434** (− 2.31)	− 0.0451*** (− 3.14)
r_1_ ≤ RD < r_2_	− 0.0113 (− 0.98)	–	−	− 0.0479*** (− 2.82)	–	− 0.0137 (− 0.93)
RD ≥ r_2_	0.0561*** (3.16)	0.0170 (0.69)	0.0806*** (2.84)	0.0223 (0.92)	0.0069 (0.34)	0.0557*** (2.77)
DL	0.0113*** (4.10)	0.0191*** (3.34)	0.0101* (1.95)	0.0075** (2.07)	0.0131*** (2.77)	0.0104*** (3.23)
ROA	− 0.0000 (− 0.01)	0.0007 (1.34)	− 0.0003 (− 0.64)	− 0.0025*** (− 2.95)	0.0005 (1.27)	− 0.0013* (− 1.69)
ROE	− 0.0000 (− 0.19)	− 0.0000 (− 0.22)	0.0000 (0.58)	− 0.0000*** (− 4.93)	− 0.0000* (− 1.68)	0.0000* (1.75)
ROL	− 0.0001 (− 0.58)	0.0006 (1.16)	0.0000 (0.54)	− 0.0004*** (− 3.47)	0.0009** (2.20)	− 0.0002 (− 1.36)
Scale	− 0.0617*** (− 11.63)	− 0.0609*** (− 5.16)	− 0.0653*** (− 6.81)	− 0.0592*** (− 8.63)	− 0.0681*** (− 6.39)	− 0.0587*** (− 9.88)
cons	2.0703*** (18.26)	2.0292*** (8.04)	2.1222*** (10.12)	2.0457*** (13.95)	2.1930*** (9.37)	1.9990*** (15.92)
R^2^	0.0291	0.0296	0.0336	0.0475	0.0342	0.0321
F	31.96***	6.95***	9.64***	32.02***	12.88***	20.91***

***, ** and * indicate the statistical significance of 1%, 5% and 10% respectively.

**Table 6:** Threshold regression results of VER on enterprise GTFP.

	Overall GTFP	Industry 1 GTFP	Industry 2 GTFP	Industry 3 GTFP	State GTFP	Non-state GTFP
RD < r_1_	− 0.0090*** (− 3.27)	− 0.0258*** (− 4.43)	0.0035 (0.79)	− 0.0081* (− 1.94)	− 0.0094** (− 2.02)	− 0.0872** (− 2.53)
r_1_ ≤ RD < r_2_	− 0.0052** (− 1.88)	–	–	− 0.0042 (− 0.97)	–	− 0.0049 (− 1.39)
RD ≥ r_2_	0.0006 (0.20)	− 0.0211*** (− 3.66)	0.0081* (1.78)	0.0019 (0.42)	− 0.0035 (− 0.78)	0.0045 (1.09)
DL	0.0127*** (4.77)	0.0235*** (4.24)	0.0072 (1.41)	0.0093*** (2.57)	0.0152*** (3.17)	0.0123*** (3.91)
ROA	0.0000 (0.01)	0.0007 (1.41)	− 0.0030 (− 0.65)	− 0.0025*** (− 2.94)	0.0005 (1.31)	− 0.0013* (− 1.62)
ROE	− 0.0000 (− 0.22)	− 0.0000 (− 0.40)	0.0000 (0.59)	− 0.0000*** (− 5.08)	− 0.0000* (− 1.73)	0.0000* (1.66)
ROL	− 0.0001 (− 0.63)	0.0005 (1.03)	0.0000 (0.54)	− 0.0004*** (− 3.49)	0.0008** (2.13)	− 0.0002 (− 1.38)
Scale	− 0.0619*** (− 11.56)	− 0.0596*** (− 5.13)	− 0.0660*** (− 6.68)	− 0.0582*** (− 8.45)	− 0.0674*** (− 6.38)	− 0.0584*** (− 9.74)
cons	2.1151*** (18.64)	2.1875*** (8.64)	2.1102*** (10.14)	2.0246*** (14.08)	2.2161*** (9.54)	2.0343*** (16.46)
R^2^	0.0300	0.0359	0.0345	0.0457	0.0358	0.0333
F	31.02***	8.50***	10.54***	33.88***	13.47***	24.47***

***, ** and * indicate the statistical significance of 1%, 5% and 10% respectively.

Table 3 and the first column in Table 4 all show that CER has a significant dual threshold impact on enterprise GTFP. If RD < 3.79, CER has no significant impact on enterprise GTFP. If 3.79 ≤ RD < 9.21, CER has a significant positive impact on enterprise GTFP. If RD ≥ 9.21, CER also has a significant positive impact on enterprise GTFP, and the impact degree are enhanced. With the increase of enterprise R&D investment, the positive impact of CER on GTFP increases. The possible reason is that higher levels of R&D investment often incentive enterprise technological innovation, which is more suitable for strict CER. That is, with the increase of enterprise R&D investment, the stricter CER, the more conducive it is to guiding and promoting enterprises to carry out green production.

Table 3 and the first column in Table 5 all show that MER has a significant dual threshold impact on enterprise GTFP. If RD < 3.30, MER has a significant negative impact on enterprise GTFP. If 3.30 ≤ RD < 8.14, MER has no significant impact on enterprise GTFP. If RD ≥ 8.14, MER has a significant positive impact on enterprise GTFP. The results indicate that the impact of MER on enterprise GTFP presents a “U-shaped” pattern. Only when enterprise R&D investment exceeds the threshold value can MER be conducive to enterprise GTFP. Therefore, when enterprises face an increasing fierce of MER, they should try to increase R&D investment, which can help enterprises cross the threshold of negative impact, and stimulate MER’s promoting effect on GTFP.

Table 3 and the first column in Table 6 all show that VER has a significant dual threshold impact on enterprise GTFP. If RD < 3.79, VER has a significant negative impact on enterprise GTFP. If 3.79 ≤ RD < 8.14, VER has a significant negative impact on enterprise GTFP but the negative effect was reduced obviously. If RD ≥ 8.14, VER has no significant impact on enterprise GTFP. This result shows that the external environmental supervision mechanism increases enterprise environmental governance cost, which has a negative impact on enterprise GTFP in the short term, but this negative impact gradually weakens and tends to be positive with the increase of enterprise R&D investment. Thus, if companies want to avoid the negative impact of VER on GTFP, they should try to increase R&D investment as much as possible and cross the threshold of negative impact.

### Heterogeneity analysis

#### Heterogeneity of industries

The sample enterprises are subdivided into high pollution industries, medium pollution industries and low pollution industries to further discuss the threshold mechanism of heterogeneous environmental regulations affecting enterprise GTFP. The results are shown in Tables 4, 5, and 6. Among them, Industry 1 represents high pollution industries, Industry 2 represents medium pollution industries, Industry 3 represents low pollution industries.

The regression results indicate that CER, MER, and VER all have threshold effects on enterprise GTFP, and the degree of impact varies across industries. Among them, CER has a more significant environmental driving effect on enterprises in highly polluting industries, which is conducive to promoting these enterprises to increase their GTFP. This also reflects that China’s current CER is moderately and effective. Overall, they have not increased the environmental governance burden on manufacturing enterprises in medium and low pollution industries, and are also effective regulations for excessive pollution of manufacturing enterprises in high pollution industries. MER increases the internal cost of enterprise pollution governance, so it has a negative impact on enterprise GTFP as a whole. VER has a stronger regulatory effect on high pollution enterprises.

#### Heterogeneity of ownership

The sample enterprises are further subdivided into state-owned and non-state-owned enterprises to discuss the heterogeneous threshold effect. The results are shown in Tables 4, 5, and 6. Among them, State represents state-owned enterprises, Non-state represents non-state-owned enterprises.

The regression results indicate that CER, MER, and VER all have threshold effects on enterprise GTFP, and the degree of impact varies across equity structure. The impact on non-state-owned manufacturing enterprises is more significant.

### Robustness tests

Robustness test is performed by two approaches. On the one hand, the Winsorizing method was made on every variable to test the robustness of research conclusions. Specifically, all continuous variables were Winsorized at the 1% and 99% levels to mitigate the potential impact of outliers on empirical results. On the other hand, threshold regression tests were re-conducted based on the dependent variable set one-period lag. The robustness test results are shown in Table 7. The results show that all variables basically maintain the same impact direction and impact trend, and pass the significance test. The research conclusions are reliable and robust.

**Table 7:** Robustness tests results.

	Sample reduction			GTFP One-period Lagging		
	GTFP (CER)	GTFP (MER)	GTFP (VER)	GTFP (CER)	GTFP (MER)	GTFP (VER)
RD < r_1_	0.0052 (0.98)	− 0.0436*** (− 4.40)	− 0.0095*** (− 3.72)	0.0076 (1.46)	0.0167 (1.50)	0.0064 (2.20)
r_1_ ≤ RD < r_2_	0.0089* (0.98)	− 0.0131 (− 1.34)	− 0.0064** (− 2.51)	0.0016 (0.31)	−	0.0035 (1.19)
RD ≥ r_2_	0.0153*** (2.92)	0.0394*** (2.47)	− 0.0020 (− 0.73)	− 0.0048 (− 0.88)	− 0.0324*** (− 2.85)	0.0000 (0.00)
DL	0.0112*** (4.72)	0.0101*** (4.21)	0.0119*** (5.02)	0.0061* (1.88)	0.0055* (1.71)	0.0059* (1.85)
ROA	− 0.0045*** (− 4.80)	− 0.0045*** (− 4.79)	− 0.0045** (− 4.81)	0.0008* (1.81)	0.0008* (1.85)	0.0008* (1.86)
ROE	0.0006 (1.44)	0.0005 (1.38)	0.0006 (1.46)	− 0.0000 (− 0.80)	− 0.0000 (− 0.90)	− 0.0000 (− 0.88)
ROL	− 0.0003 (− 1.49)	− 0.0003 (− 1.28)	− 0.0003 (− 1.47)	0.0000 (0.51)	− 0.0000** (− 2.38)	0.0000 (0.53)
Scale	− 0.0597*** (− 12.79)	− 0.0597*** (− 12.95)	− 0.0586*** (− 12.59)	− 0.0149** (− 2.18)	− 0.0163** (− 2.38)	− 0.0161** (− 2.34)
cons	1.9806*** (20.09)	2.0466*** (20.90)	2.0671*** (21.43)	0.9827*** (6.44)	1.0461*** (6.98)	1.0124*** (6.87)
R^2^	0.0428	0.0435	0.0439	0.0098	0.0081	0.0094
F	42.16***	44.19***	45.48***	9.80***	8.16***	8.68***

***, ** and * indicate the statistical significance of 1%, 5% and 10% respectively.

## Conclusions and limitations

### Conclusions and policy implications

Based on the panel data of 1220 Chinese manufacturing listed companies from 2011 to 2020, this paper uses threshold regression model to examine the impact of heterogeneous environmental regulations on enterprise GTFP. Three main conclusions are drawn. (1) Heterogeneous environmental regulation has a double threshold impact on enterprise GTFP. Specifically, CER has a significant positive impact on enterprise GTFP, but the degree of impact decreases. MER has a significant “U-shaped” impact on enterprise GTFP. VER has a significant negative decreasing influence on enterprise GTFP. (2) Enterprise R&D investment plays a threshold role in the impact of heterogeneous environmental regulations on enterprise GTFP. The “Follow Cost” and “Porter Hypothesis” effects act at different stages. And these findings remain valid after a series of robustness tests. (3) There are industry and ownership differences in the impact of heterogeneous environmental regulations on GTFP. In general, environmental regulations have a more significant impact on enterprises in highly polluting industries and non-state-owned enterprises.

These conclusions have valuable policy implications for formulating flexible environmental regulations and promoting Chinese enterprises low-carbon development. Firstly, the empirical results show that environmental regulations are not the stricter the better. On the one hand, the environmental effects motivated by environmental regulations show significant dual threshold effect, and the trend of MER is most obviously. On the other hand, the environmental effects motivated by environmental regulations show industry and ownership differences. Thus, the government should develop a flexible environmental regulation system and prioritize the use of market-incentive environmental regulation measures. Secondly, the empirical results show that enterprises R&D investment determine the influence direction and degree. Therefore, manufacturing enterprises should rely on more R&D investment to decrease the “obstructive effects of environmental regulations”, and achieve green, low-carbon, and sustainable development while improving enterprise productivity simultaneously.

### Limitations and future research

This paper still has some limitations which may also be directions for future research. Firstly, in view of limited data, “enterprises industrial wastes data” used in this study is to quantify the industrial wastewater, SO_2_ and industrial smoke emissions at the city level to the enterprise level on a year-on-year basis through the “proportion of total output value of enterprises”. In future studies, data algorithms can be further improved to evaluate the effectiveness of heterogeneous environmental regulations on enterprises green growth. Secondly, this article mainly explores the threshold effect of heterogeneous environmental regulations on enterprise GTFP. Nowadays, some studies have pointed out that there is a two-way dynamic relationship between heterogeneous environmental regulations and green development^53^, and the environmental effects motivated by environmental regulations have spillover effects^7^. Therefore, further research can explore these aspects in depth. Thirdly, digitalization is booming worldwide attention, which has brought both opportunities and challenges to enterprises green development. Although enterprise digital level has been considered as a controllable variable in this article, we didn’t mainly discuss its effects. In the future, researches could conduct in-depth researches on the impact of enterprise digital level as a core variable on enterprise GTFP.

## Data Availability

Data will be made available on request.

## References

[1] Dai J, Chen B, Hayat T, Alsaedi A, Ahmad B (2015). Sustainability-based economic and ecological evaluation of a rural biogas-linked agro-ecosystem. Renew. Sustain. Energy Rev..

[2] Lu WX, Wu HC, Geng SS (2021). Heterogeneity and threshold effects of environmental regulation on health expenditure: Considering the mediating role of environmental pollution. J. Environ. Manag..

[3] Wang AL, Hu S, Lin BQ (2021). Can environmental regulation solve pollution problems? Theoretical model and empirical research based on the skill premium. Energy Econ..

[4] Lu WX, Wu HC, Yang SJ, Tu YL (2022). Effect of environmental regulation policy synergy on carbon emissions in China under consideration of the mediating role of industrial structure. J. Environ. Manag..

[5] Zhang DY, Zhao R, Ji Q (2019). Green innovation and firm performance: Evidence from listed companies in China. Resour. Conserv. Recycl..

[6] Romer P (1990). Endogenous technological change. J. Political Econ..

[7] Ahmed EM (2020). Modelling green productivity spillover effects on sustainability. World J. Sci. Technol. Sustain. Dev..

[8] Abid N, Ahmad F, Aftab J, Razzaq A (2023). A blessing or a burden? Assessing the impact of Climate Change Mitigation efforts in Europe using Quantile Regression Models. Energy Policy.

[9] Jaffe AB, Stavins RN (1995). Dynamic incentives of environmental regulations: The effects of alternative policy instruments on technology diffusion. J. Environ. Econ. Manag..

[10] Hamamoto M (2006). Environmental regulation and the productivity of Japenese manufacturing industries. Resour. Eng. Econ..

[11] Zhao X, Sun B (2016). The influence of Chinese environmental regulation on corporation innovation and competitiveness. J. Clean. Prod..

[12] Jaffe AB, Palmer K (1997). Environmental regulation and innovation: A panel data study. Review Econ. Stat..

[13] Fernando Y, Jabbour CJC, Wah WX (2019). Pursuing green growth in technology firms through the connections between environmental innovation and sustainable business performance: Does service capability matter?. Resour. Conserv. Recycl..

[14] Christiansen GB, Haveman RH (1981). The contribution of environmental regulations to slowdown in productivity growth. J. Environ. Econ. Manag..

[15] Xie R, Yuan Y, Huang J (2017). Different types of environmental regulations and heterogeneous influence on “green” productivity: Evidence from China. Ecol. Econ..

[16] Jaffe AB, Peterson SR, Portney PR, Stavins PN (1994). Environmental regulation and the competitiveness of US manufacturing: what does the evidence tell us. J. Econ. Lit..

[17] Porter ME (1991). Towards a dynamic theory of strategy. Strateg. Manag. J..

[18] Boyd GA, Tolley G, Pang J (2002). Plant level productivity, efficiency, and environmental performance of the container glass industry. Environ. Resour. Econ..

[19] Leeuwen G, Mohnen P (2017). Revisiting the Porter Hypothesis: An empirical analysis of green innovation for the Netherlands. Econ. Innov. New Technol..

[20] Rens G, Li XL, Yuan BL (2018). The effect of three types of environmental regulation on eco-efficiency: A crossregion analysis in China. J. Clean. Prod..

[21] Heuser, C., Mattoo, A. Services Trade and Global Value Chains in *Policy Research Working Paper* (World Bank) No. 8126 (Washington, DC, 2017).

[22] Li R, Ramanathan R (2018). Exploring the relationships between different types of environmental regulations and environmental performance: evidence from China. J. Clean. Prod..

[23] Guo LL, Qu Y, Wu CY, Wang XL (2018). Identifying a pathway towards green growth of Chinese industrial regions based on a system dynamics approach. Resour. Conserv. Recycl..

[24] Van LG, Mohnen P (2017). Revisiting the Porter hypothesis: An empirical analysis of green innovation for The Netherlands. Econ. Innovat. N. Technol..

[25] Ma R, Abid N, Yang S, Ahmad F (2020). From crisis to resilience: strengthening climate action in OECD countries through environmental policy and energy transition. Environ. Sci. Pollut. Res..

[26] Cui SN, Wang YQ, Zhu ZW, Zhu ZH, Yu CY (2022). The impact of heterogeneous environmental regulation on the energy eco-efficiency of China’s energy-mineral cities. J. Clean. Prod..

[27] Fang Y, Shao Z (2022). Whether green finance can effectively moderate the green technology innovation effect of heterogeneous environmental regulation. Int. J. Environ. Res. Public Health.

[28] Luo YS, Salman M, Lu ZN (2021). Heterogeneous impacts of environmental regulations and foreign direct investment on green innovation across different regions in China. Sci. Total Environ..

[29] Wang LP, Long Y, Li C (2022). Research on the impact mechanism of heterogeneous environmental regulation on enterprise green technology innovation. J. Environ. Manag..

[30] Yi M, Fang XM, Wen L, Guang FT, Zhang Y (2019). The heterogeneous effects of different environmental policy instruments on green technology innovation. Int. J. Environ. Res. Public Health.

[31] Song Y, Yang T, Zhang M (2019). Research on the impact of environmental regulation on enterprise technology innovation—An empirical analysis based on Chinese provincial panel data. Environ. Sci. Pollut. Res..

[32] Wang X, Chai Y, Wu W, Khurshid A (2023). The empirical analysis of environmental regulation’s spatial spillover effects on green technology innovation in China. Int. J. Environ. Res. Publ. Health.

[33] Bu ML, Qiao ZZ, Liu BB (2020). Voluntary environmental regulation and firm innovation in China. Econ. Modell..

[34] Gray WB, Shadbegian RJ (2003). Plant vintage, technology, and environmental regulation. J. Environ. Econ. Manag..

[35] Feng M, Chen YQ (2023). Impacts of heterogenous environmental regulations on green innovation of new energy firms: empirical evidence from China. J. Environ. Stud..

[36] Wirth C, Chi J, Young M (2013). The economic impact of capital expenditures: Environmental regulatory delay as a source of competitive advantage?. J. Bus. Financ. Account..

[37] Jeffrey C, Perkins JD (2014). The Relationship between energy taxation and business environmental protection expenditures in the European Union. Int. J. Account..

[38] Wang LP, Li C (2022). Experimental research on green technology innovation behavior based on voluntary contribution mechanism. J. Environ. Prot. Ecol..

[39] Berman E, Bui LTM (2001). Environmental regulation and productivity: Evidence from oil refineries. Rev. Econ. Stat..

[40] Jia K, Chen S (2019). Could campaign-style enforcement improve environmental performance? Evidence from China’s central environmental protection inspection. J. Environ. Manag..

[41] Zhu XH, Zuo XG, Li HL (2021). The dual effects of heterogeneous environmental regulation on the technological innovation of Chinese steel enterprises—Based on a high-dimensional fixed effects model. Ecolo. Econ..

[42] Sun ZY, Wang XP, Liang C, Cao F, Wang L (2021). The impact of heterogeneous environmental regulation on innovation of high-tech enterprises in China: Mediating and interaction effect. Environ. Sci. Pollut. Res..

[43] Tu ZG, Shen RJ (2015). Can emissions trading scheme achieve the porter effect in China?. Econ. Res..

[44] Zhong SH, Xiong YJ, Xiang GC (2021). Environmental regulation benefits for whom? Heterogeneous effects of the intensity of the environmental regulation on employment in China. J. Environ. Manag..

[45] Saunila M, Ukko J, Rantala T (2018). Sustainability as a driver of green innovation investment and exploitation. J. Clean. Prod..

[46] Klewitz J, Hansen EG (2014). Sustainability-oriented innovation of SMEs: A systematic review. J. Clean. Prod..

[47] Tone K (2001). A slacks-based measure of efficiency in data envelopment analysis. Eur. J. Oper. Res..

[48] Tone K, Tsutsui M (2010). An epsilon-based measure of efficiency in DEA: A third pole of technical efficiency. Eur. J. Oper. Res..

[49] Guo R, Yuan YJ (2020). Different types of environmental regulations and heterogeneous influence on energy efficiency in the industrial sector: Evidence from Chinese provincial data. Energy Policy.

[50] Wang C, Zhang YJ (2020). Does environmental regulation policy help improve green production performance? Evidence from China’s industry. Corp. Soc. Responsib. Environ. Manag..

[51] Li HL, Zhu XH, Chen JY, Jiang FT (2019). Environmental regulations, environmental governance efficiency and the green transformation of China’s iron and steel enterprises. Ecol. Econ..

[52] Liu Y, Li Z, Yin X (2018). The effects of three types of environmental regulation on energy consumption—Evidence from China. Environ. Sci. Pollut. Res..

[53] Zhang N, Deng J, Ahmad F, Draz MU, Abid N (2023). The dynamic association between public environmental demands, government environmental governance, and green technology innovation in China: Evidence from panel VAR model. Environ. Dev. Sustain..

[54] Tang K, Qiu Y, Zhou D (2020). Does command-and-control regulation promote green innovation performance? Evidence from China’s industrial enterprises. Sci. Total Environ..

[55] You D, Zhang Y, Yuan B (2019). Environmental regulation and firm eco-innovation: Evidence of moderating effects of social decentralization and political competition from listed Chinese industrial companies. J. Clean. Prod..

[56] Liang H, Shi C, Abid N, Yu Y (2023). Are digitalization and human development discarding the resource curse in emerging economies?. Res. Policy..

[57] Hansen BE (1999). Threshold effects in non-dynamic panels: Estimation, testing and inference. J. Econo..

